# Myositis in Germany: epidemiological insights over 15 years from 2005 to 2019

**DOI:** 10.1186/s42466-022-00226-4

**Published:** 2022-12-29

**Authors:** Marc Pawlitzki, Laura Acar, Lars Masanneck, Alice Willison, Liesa Regner-Nelke, Christopher Nelke, Helmut L’hoest, Ursula Marschall, Jens Schmidt, Sven G. Meuth, Tobias Ruck

**Affiliations:** 1 Department of Neurology, Heinrich Heine University Düsseldorf, Moorenstraße 5, 40225 Düsseldorf, Germany; 2 BARMER, Wuppertal, Germany; 3 Hasso-Plattner-Institut, Potsdam, Germany; 4 Department of Neurology and Pain Therapy, Immanuel Clinic Rüdersdorf, University Hospital of the Brandenburg Medical School - Theodor Fontane, Rüdersdorf Berlin, Germany; 5 Faculty of Health Sciences Brandenburg, Brandenburg Medical School - Theodor Fontane, Rüdersdorf Berlin, Germany; 6 Department of Neurology, Neuromuscular Centre, University Medical Centre Göttingen, Göttingen, Germany

## Abstract

**Background:**

The medical care of patients with myositis is a great challenge in clinical practice. This is due to the rarity of these disease, the complexity of diagnosis and management as well as the lack of systematic analyses.

**Objectives:**

Therefore, the aim of this project was to obtain an overview of the current care of myositis patients in Germany and to evaluate epidemiological trends in recent years.

**Methods:**

In collaboration with BARMER Insurance, retrospective analysis of outpatient and inpatient data from an average of approximately 8.7 million insured patients between January 2005 and December 2019 was performed using ICD-10 codes for myositis for identification of relevant data. In addition, a comparative analysis was performed between myositis patients and an age-matched comparison group from other populations insured by BARMER.

**Results:**

45,800 BARMER-insured individuals received a diagnosis of myositis during the observation period, with a relatively stable prevalence throughout. With regard to comorbidities, a significantly higher rate of cardiovascular disease as well as neoplasm was observed compared to the control group within the BARMER-insured population. In addition, myositis patients suffer more frequently from psychiatric disorders, such as depression and somatoform disorders. However, the ICD-10 catalogue only includes the specific coding of “dermatomyositis” and “polymyositis” and thus does not allow for a sufficient analysis of all idiopathic inflammatory myopathies subtypes.

**Conclusion:**

The current data provide a comprehensive epidemiological analysis of myositis in Germany, highlighting the multimorbidity of myositis patients. This underlines the need for multidisciplinary management. However, the ICD-10 codes currently still in use do not allow for specific analysis of the subtypes of myositis. The upcoming ICD-11 coding may improve future analyses in this regard.

## Introduction

The medical management of myositis remains challenging due to its rarity and heterogeneity. In particular, the diagnosis is often delayed for years following initial disease manifestation and usually requires extensive investigation including clinical examination, electrophysiology, antibody diagnostics, magnetic resonance imaging and muscle biopsy [1–3]. The situation is further complicated by the fact that, despite appropriate diagnostic investigation, no uniform diagnostic criteria are available and systematic analyses therefore often include only a small number of patients [4, 5].

With regard to drug therapy, there is usually no standardized approach despite recommendations to this effect, since data regarding the efficacy and safety of classical immunosuppressants are mostly based on retrospective study results. Various biologics are also increasingly being prescribed on an individual basis as a therapeutic trial due to the lack of randomized, controlled studies (RCTs) [6]. Due to the potential involvement of several organ systems, patients are also cared for by numerous medical specialties, so standardized procedures can usually only be established within cooperation networks or interdisciplinary case conferences [7, 8].

In order to adequately cover the need for care in the future and to establish a uniform diagnostic and therapeutic approach, it is necessary to collect demographic, clinical and therapeutic trends in an appropriately large patient population. To date, however, only a few case series and registry data exist that provide insights into the provision of care for myositis in Germany [7]. In most cases, these are analyses of patients who are treated in specialist centres or practices. Nationwide data from routine care are still lacking.

The aim of the current study was therefore to provide an overview of the real healthcare situation in Germany with the help of insurance data from BARMER Health Insurance and to capture epidemiological trends in recent years, which could ultimately form the basis for future diagnostic and therapeutic approaches. The health insurance data provided by BARMER, the second largest health insurance company in Germany, cover the outpatient and inpatient medical care of currently up to 8.7 million insured persons (10.5% of the German population), with more than 154 million insured years between January 1, 2005 and December 31, 2019. This makes it possible to perform comparative analyses between myositis patients and an age-matched comparison group from the other insured individuals within the BARMER cohort. Age- and sex-standardized extrapolation of data for comparison to the general population in Germany was performed using data from the Federal Statistical Office [9, 10].

## Methods

In collaboration with BARMER, outpatient and inpatient insured data from January 2005 to December 2019 were retrospectively analysed using ICD-10 codes for myositis (M33.0: Juvenile dermatomyositis; M33.1: Other dermatomyositis; M33. 2: Polymyositis; M33.9: Dermatomyositis-polymyositis, unspecified; M60.1: Interstitial myositis, M60.8: Other myositis; M60.9: Myositis, unspecified; G72.4: Inflammatory myopathy, not elsewhere classified). Only patients with the indicator of a "confirmed" diagnosis were included. The relative frequencies of myositis per 100,000 insured persons for age and sex were calculated. A distinction was made between dermatomyositis (DM; M33.0, M33.1, M33.9), polymyositis (PM; M33.2), interstitial myositis (M60.1), and other myositis (G72.4, M60.8, M60.9). Overlap syndromes (M35.1) were deliberately not considered in order to include only patients who also showed muscle involvement, where possible. If both a specific (DM, PM, interstitial myositis) and a non-specific diagnosis (other myositis) was coded, the patient was assigned to a group according to the specific diagnosis. The prevalence data were standardized according to age and sex and extrapolated using general German population data from 2019. With regard to incidence calculation, patient datapoints were included if the corresponding ICD code was documented for the first time at a given time point, but not in the 4 years prior to that. This ensured that, as far as possible, only first diagnoses were included. To determine the age at first diagnosis, patients with a diagnosis coded for the first time in 2015 were analysed, as this—considering data collection was from 2005 to 2019—provided an observation period of approximately 10 years to exclude the possibility of a previous diagnosis of myositis. Patient records with periods without insurance were excluded. In addition, the following ICD-10 codes were used to identify neoplasms: C00.- to C97.-; psychiatric disorders: F32.-, F.33.-, F41.-, F45.-; and infectious diseases: A16.-, A40.-, A41.-, J12.- to J18.- as relevant comorbidities in the patient population. Again, only confirmed diagnoses were included. The calculated rates were compared with a sex- and age-matched comparison group from the BARMER control population. The most frequent reasons for hospitalization of myositis patients were also examined. Here, the ICD-10 codes of the primary diagnoses leading to hospitalization of myositis patients were evaluated and compared with the corresponding rates from the BARMER control population.

For the identification of disease-associated symptoms potentially occurring prior to the diagnosis of myositis, patients with a first diagnosis in 2015 were considered to ensure a sufficiently long observation period from 2005 to 2015. Regarding the analysis of outpatient prescriptions of immunotherapies, the following drugs with corresponding ATC codes were included (immunosuppressants: azathioprine L04AX01, cyclophosphamide: L01AA01, ciclosporin: L04AD01, hydroxychloroquine: P01BA02, methotrexate: L04AX03, mycophenolic acid: L04AA06; sirolimus: L04AA10, tacrolimus: L04AD02; biologics: rituximab: L01XC02; prednisolone: H02AB06, prednisone: H02AB07).

## Results

**Fig. 1:**
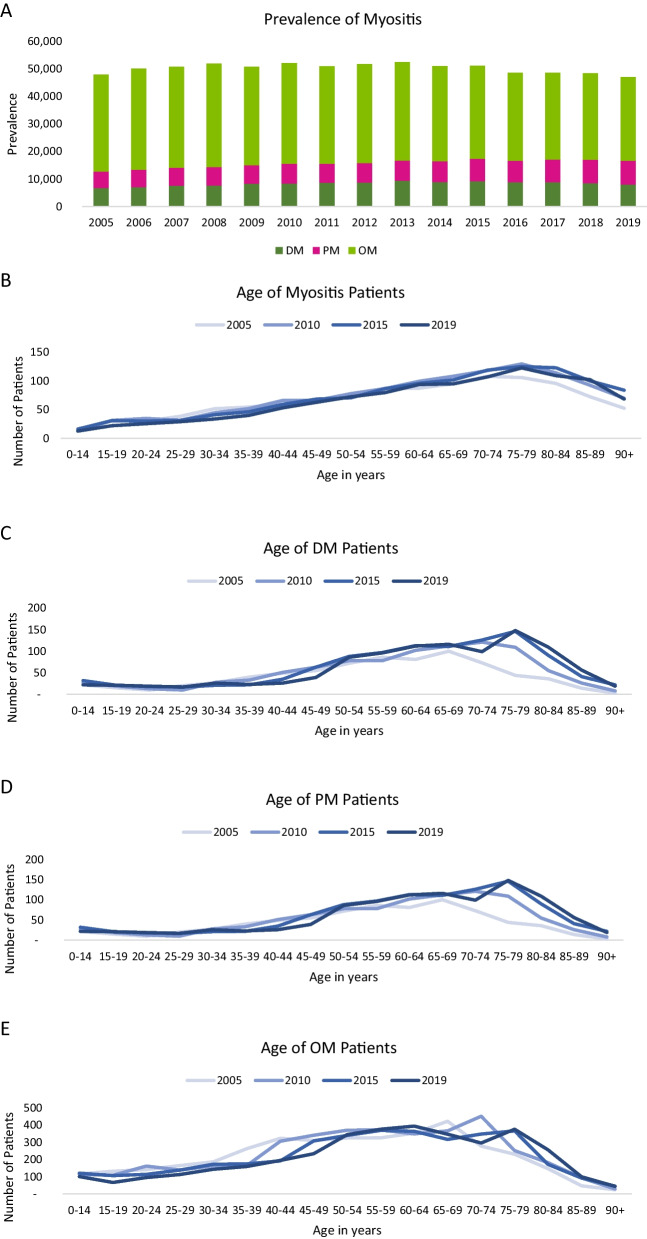
Prevalence and age distribution of myositis patients in Germany. **A** Shown are the standardized absolute values of myositis patients in the observation period from 2005 to 2019 in Germany. **B–E** Age distribution of the total myositis cohort. (**B**) and the myositis subgroups (**C–E**) and the chronological trend during the observation period from 2005 to 2019 per 100,000 insured persons. *DM* dermatomyositis, *IM* interstitial myositis, *PM* polymyositis, *OM* other myositis

In total, approximately 45,800 BARMER-insured individuals were coded with a diagnosis of “myositis” during the study period. There was no relevant increase in the prevalence of myositis from 2005 to 2019 (60.27/100,000 vs. 64.57/100,000). The prevalence of “other myositis” diagnosis decreased by 8% from 2005 to 2019 (43.31/100,000 vs. 39.67/100,000), whereas the prevalence of “DM” (+ 32%) and “PM” (+ 80%) increased (Table 1). Sex- and age-standardized extrapolation demonstrates approximately 8,000 patients with “DM”, 8,600 patients with “PM”, 820 patients with “interstitial myositis”, and approximately 30,500 patients with “other myositis” in 2019 (Fig. 1A). With regard to the age distribution of myositis and the individual diagnostic groups, an increasing frequency of myositis in older age is observed, with a noticeable upward trend from the age of 40 and a clear peak between the ages of 70 and 80 (Fig. 1B–E). For the “interstitial myositis” group, further data analysis was not possible due to the small number of cases.

**Table 1:** Development of the prevalence rates (per 100,000) in the observation period from 2005 to 2019

Year	Total	DM	PM	IM	OM
2005	60.3	8.6	6.9	1.5	43.3
2006	64.3	8.9	7.6	2.3	45.4
2007	65.7	9.7	8.1	2.3	45.5
2008	66.5	9.9	8.5	1.5	46.7
2009	65.5	10.6	8.7	1.5	44.7
2010	67.5	10.9	9.3	1.3	46.0
2011	65.9	11.2	9.1	1.3	44.3
2012	66.8	11.5	9.3	1.1	44.8
2013	68.5	12.4	9.8	1.2	45.1
2014	66.9	11.8	10.1	1.2	43.9
2015	67.8	12.3	11.1	1.2	43.2
2016	65.2	12.0	10.9	1.3	40.9
2017	65.8	12.3	11.6	1.4	40.5
2018	65.5	11.8	12.0	1.3	40.4
2019	64.6	11.3	12.5	1.1	39.7

*DM* dermatomyositis, *IM* interstitial myositis, *PM* polymyositis, *OM* other myositis

**Fig. 2:**
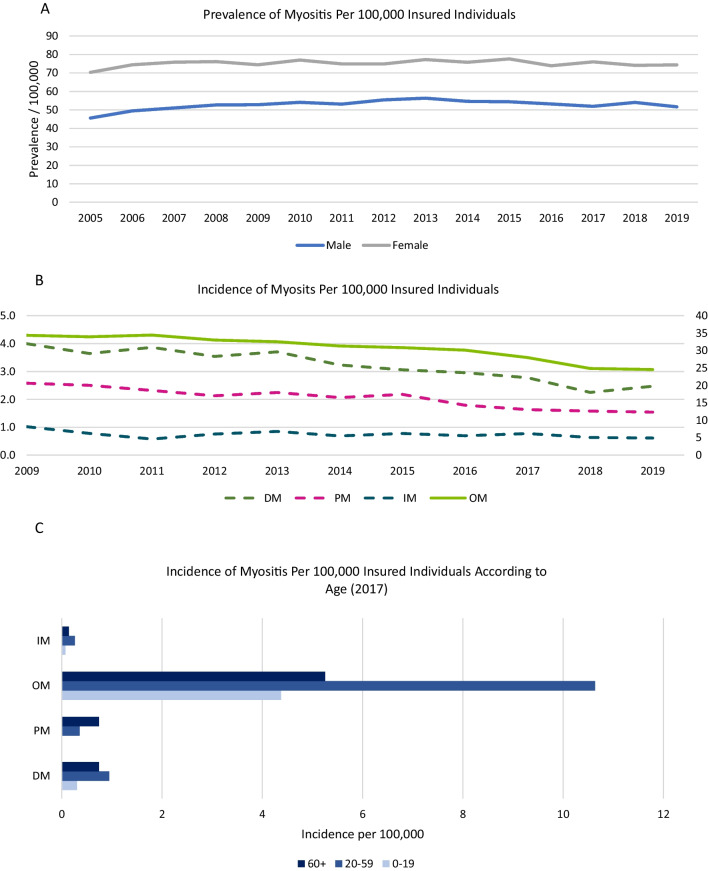
Sex distribution, incidence and the dynamics of these factors over time. **A** Sex distribution of myositis patients and chronological trend in the observation period from 2005 to 2019 per 100,000 insured persons. **B** Incidences of the myositis subgroups and trend over time per 100,000 insured persons. Left Y-axis: rates of DM, PM, IM; Right Y-axis: rates of OM. **C** New cases per 100,000 insured persons, age group and myositis subgroup. *DM* dermatomyositis, *IM* interstitial myositis, *PM* polymyositis, *OM* other myositis

Women were affected more often overall and in each of the diagnostic groups studied (2019: 74.4/100,000 women vs. 51.7/100,000 men), In male patients, there was an increase of about 13.3% from 2005 to 2019 (45.6/100,000 men vs. 51.7/100,000 men), while the prevalences in female patients increased by about 5.8% in the same period (70.3/100,000 women vs. 74.4/100,000 women) (Fig. 2A). Among the myositis groups, there was a significantly higher proportion of female patients in “DM” (2019: 14.3 vs. 7.3), PM (2019: 14.9 vs. 9.2), and “other myositis” (2019: 43.7 vs. 34.3). Regarding the diagnosis groups, there was a significantly higher proportion of female patients in “DM” (2019: 14.3 vs. 7.3), “PM” (2019: 14.9 vs. 9.2) and “other myositis” (2019: 43.7 vs. 34.3). Annual incidences have tended to decrease in recent years for all ICD-10 diagnoses studied. In particular, a significant reduction over the past 5 years is observed in “PM” (2009: 2.6/100,000 vs. 2019: 1.5/100,000) (Fig. 2B). The age at first diagnosis is mostly above the age of 60, although this differs significantly for the individual diagnostic groups. In particular, “PM” is diagnosed more frequently in people over 60 years of age compared to “DM”, “interstitial myositis” and “other myositis”, which are more likely to be diagnosed between the ages of 20 and 59 (Fig. 2C).

**Fig. 3:**
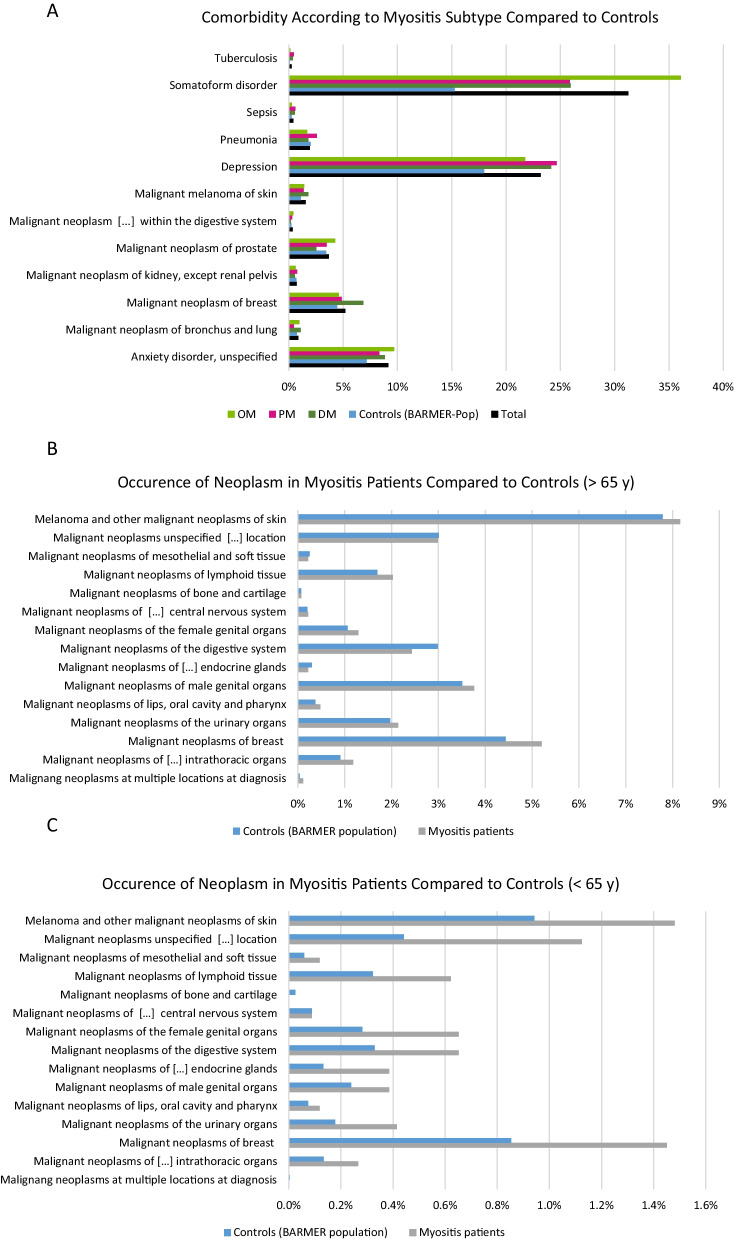
Comorbidities of myositis patients. **A** Relative frequencies of comorbidities based on ICD-10 codes for the entire myositis cohort (total), the myositis subgroups (DM = dermatomyositis, PM = polymyositis, OM = other myositis) and the control population in the BARMER cohort according to sex. **B** Relative frequencies of neoplasms in the entire myositis cohort over the age of 65 compared to the control population in the BARMER cohort. **C** Relative frequencies of neoplasms in the entire myositis cohort under the age of 65 compared to the control BARMER cohort

Symptoms typically coded before myositis diagnosis include skin conditions (unguis incarnatus 3%, nail dystrophy 2%) and cardiac and pulmonary symptoms (dyspnoea 8%, heart failure 2%). Regarding comorbidities, psychiatric disorders, such as depression, anxiety disorder, and especially somatoform disorders are frequently observed in both female and male patients. For somatoform disorders, there is a significantly higher percentage compared to the BARMER control population (female: 34.0% vs. 17.8%, male: 25.5% vs. 10.7%) (Fig. 3A). Among the myositis groups, a significantly higher proportion of somatoform disorders was found in patients with “other myositis” (female: 39.4%, male: 29.9%). Of all myositis insurants from 2005 to 2019 (45,800), 11,400 had at least one confirmed neoplasia as diagnosis. This represents about 25% of patients. Neoplasia was not significantly more frequent in myositis patients over 65 years of age compared to the BARMER control population (Fig. 3B). However, a higher number of neoplasms was observed in patients under the age of 65 compared to the control BARMER cohort. In particular, the rate of breast carcinomas (1.45% vs. 0.85%) and neoplasms of the skin (1.48% vs. 0.94%) were slightly increased in the myositis groups (Fig. 3C).

**Fig. 4:**
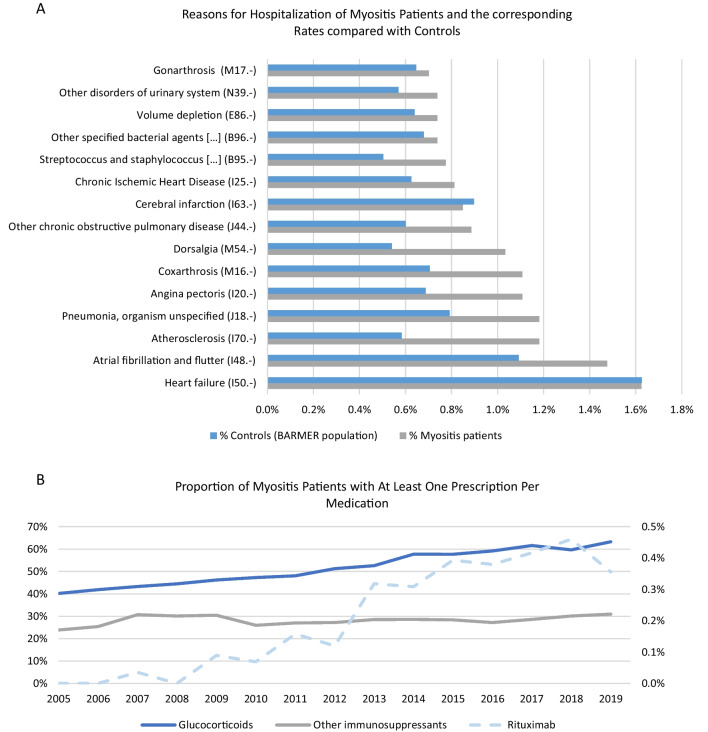
Hospitalisation rate and treatment trends of myositis patients. **A** Analysis of the reasons for inpatient admission of myositis patients compared to the control group of the BARMER cohort (BARMER population) with relative frequencies. **B** Proportion of patients on glucocorticoid and other immunosuppressant therapy and/or rituximab therapy with at least one prescription per year

Cardiovascular disease represents the most frequent reason for admission for inpatient hospital treatment in female and male myositis patients aged 65 and older and, although rare overall, occurs more frequently compared to the respective sex- and age-matched BARMER control population (atrial fibrillation/atrial flutter female: 1.5% vs. 1.1%, male: 1.5% vs. 1.2%; angina pectoris female: 0.8% vs. 0.5%, male: 1.7% vs. 1.0%; chronic ischemic heart disease male: 1.9% vs. 1.1%; heart failure female: 1.8% vs. 1.5%) (Fig. 4A). There was a high number of glucocorticoid prescriptions that increased slightly in frequency. The outpatient prescription of rituximab also increased, whereas the number of other immunosuppressant prescriptions remained constant over time (Fig. 4B).

## Discussion

Due to the rarity and complexity of myositis, large case numbers are needed to understand the current provision of healthcare so that the need for altered or additional medical investigations and therapies can be identified in a patient-oriented manner. This study allowed the analysis of relevant data from more than 45,800 patients with myositis. In addition, using established data for extrapolation, relevant epidemiological information could be derived for the entire population of Germany, which highlighted the significant medical challenges in the treatment of patients with myositis.

The evaluated data indicate a higher prevalence of myositis than was previously assumed [11]. However, previous work primarily considers idiopathic inflammatory myopathies (IIM), particularly PM and DM [12–16]. Even for the much more clearly defined ICD-10 codes, higher prevalence of myositis was documented on the basis of the available data compared with earlier work from North America, Europe, and Asia [12, 14, 15]. As geographic differences have not been demonstrated in systematic reviews so far, our data may indicate an overall underestimated prevalence of myositis. However, due to the ICD codes used, secondary myositis syndromes were also classified as “other myositis”, as there are overlaps in the coding (e.g., G72.0 Drug-induced myopathy). However, in light of the rarity of these secondary causes [17–19], it can be assumed that the present data are only slightly influenced by this classification of “other myositis”. The increasing prevalence of myositis in older age shown in recent years may be explained, regarding the discrepancy with previous work, by increasing life expectancy [11]. However, in terms of incidences, there has been a slight decrease in diagnosis in recent years, although the corresponding rates are still higher than shown in previous work [11]. It is striking that the rate of PM diagnoses in particular is decreasing noticeably, which may be due to the fact that this diagnosis is recently more likely to be regarded as a diagnosis of exclusion in IIM on the basis of new diagnostic criteria [20, 21]. With regard to the question of potential early symptoms of myositis, skin conditions as well as cardiac and pulmonary symptoms were found, but these were nonspecific. In contrast, early neurological symptoms such as myalgias, paresis or pain do not seem to be typical coded early symptoms.

Concerning comorbidities, a high rate of neoplasms in myositis patients is of particular interest. Particularly in patients under the age of 65, there is an increased incidence of gynaecological, gastrointestinal, and skin neoplasms, which confirms previous analyses, especially in the context of a diagnosis of PM or DM [22–24]. The cumulative rate of malignancy is in line with previous analyses in IIM patients and emphasizes the need for appropriate screening in this patient population after diagnosis [15, 25–27].

Furthermore, it is apparent that mainly psychiatric comorbidities occur in myositis patients. Although an increased prevalence of depressive disorder in chronic diseases is known, there are no systematic studies for myositis patients yet available. Only in DM has a small study shown that just under half of those affected suffer from depression and/or an anxiety disorder. More importantly, almost 1/3 of these patients did not receive appropriate specialized care [28]. Given the already significant reduction in patient quality of life due to myositis, our data highlight the need for increased attention to relevant comorbidities in clinical practice. In addition, more than every fourth myositis patient is diagnosed with a somatoform disorder. This also illustrates the high incidence of this comorbidity among myositis patients and in particular in the other myositis group. Since myopathic symptoms such as myalgias of unknown aetiology are probably also classified as “other myositis” in everyday clinical practice, the increased incidence of somatoform disorder could indicate this should be considered as a differential diagnosis. A targeted diagnostic and therapeutic psychosomatic assessment should therefore be considered. It should be noted that concomitant rheumatologic diseases, which frequently affect IIM patients [29], may be underrepresented in this analysis due to the exclusion of overlap syndromes in the selection of ICD-10 codes.

Lastly, an analysis of the currently used medication in myositis patients demonstrated, not unexpectedly, that glucocorticoids were commonly used as a long-term medication. The rate of outpatient prescription of immunosuppressants appears to have been constant over the past few years. The fact that long-term glucocorticoids still seem to be necessary for the treatment of patients despite the high usage of immunosuppressants underlines the current need for further treatment options for myositis patients [6]. Despite only outpatient prescriptions being considered with regard to the use of rituximab, there was also a trend towards using this specific immunotherapy. Several studies indicate that under rituximab disease stability, in particular of the extramuscular symptoms, can be achieved [30, 31]. However, rituximab is used off-label due to a lack of conclusive RCTs in this area. Accordingly, it can be assumed that rituximab is prescribed cautiously in the outpatient setting. Due to the favourable results of new immunotherapies in myositis patients, significant changes to treatment are to be expected in the future [32].

However, this study has several limitations. First, we did not perform statistical comparisons between the cohorts due to the small number of patients per subgroup. More extensive analyses with statistical significance tests and propensity score matching for the formation of a reference population are planned for future work. Second,, it should be noted that, due to the ICD-10 codes, more specific analyses of subtypes, such as anti-synthetase syndrome or inclusion body myositis, could not be performed. The latter should be coded as G72.4 according to the recommendation of the German Society of Neurology [33]. Whether this is will be implemented in clinical practice is questionable. In all probability, it can be assumed that these diseases will be coded as “other myositis” or even as “PM”, since the term "polymyositis", similar to "polyneuropathy", serves a descriptive function. Furthermore, it remains unclear which conditions are masked behind the ICD-10 diagnosis of “interstitial myositis”, as these are not used in previous or current diagnostic criteria.

Therefore, the ICD-10 codes used do not reflect the current scientific knowledge and additionally complicate in-depth analyses regarding specific early symptoms, comorbidities, and treatment. The ICD-11 coding in effect since the beginning of the year allows at least the specific coding of inclusion body myositis and a corresponding further breakdown of DM into juvenile and adult forms (Table 2) [34]. In addition to the unspecific and incomplete ICD-10 coding, however, it should be mentioned that the large number of different diagnostic criteria makes uniform coding difficult as a part of routine clinical practice [35]. This also explains, for example, the high number of PM cases, as recent studies suggest that this entity should represent the rarest form of myositis [20]. Furthermore, it cannot be excluded that patients received a myositis diagnosis without fulfilling the appropriate diagnostic criteria. A study by Dobloug et al. illustrates that most of the myositis patients included in the analysed cohort (approximately 90%) did not meet the diagnostic criteria, which could explain the significantly higher prevalence in our data [15]. In other recent epidemiologic work, ICD-10 codes were also ostensibly used as the basis of the analyses [11]. In this respect, the future increased use of the ICD-11 code with at least a rudimentary further breakdown of myositis diagnoses could enable specific findings for corresponding subtypes and additionally clarify the complexity of myositis subgroups and their complex management in clinical practice.

**Table 2:** Comparison of ICD-10 and ICD-11 coding for myositis with a focus on idiopathic inflammatory myopathies

ICD-10-code	ICD-11-code
**M33.- Dermatomyositis-polymyositis**	**4A41 Idiopathic inflammatory myopathy**
**M33.0** juvenile dermatomyositis **M33.1** other dermatomyositis	**4A41.0** Dermatomyositis ***4A41.00**** adult dermatomyositis* ***4A41.01**** juvenile dermatomyositis* ***4A41.0Z**** Dermatomyositis, unspecified*
**M33.2** Polymyositis **M33.9** Dermatomyositis-polymyositis, unspecified	**4A41.1** Polymyositis ***4A41,10**** Juvenile polymyositis* ***4A41,11**** Paraneoplastic polymyositis* ***4A41,1**** other unspecified polymyositis* ***4A41,1Z**** Polymyositis, unspecified*
**M60.1** Interstitial myositis **M60.8** Other myositis **G72.4** Inflammatory myopathy, not elsewhere classified	**4A41,2** Inclusion body myopathy ***4A41,20**** Inflammatory inclusion body myopathy* ***4A41,21**** Noninflammatory inclusion body myopathy* ***4A41,2Z**** Inclusion body myopathy, unspecified*
**M60.9** Myositis, not elsewhere classified	**4A41. Y** Other specified idiopathic inflammatory myopathy **4A41. Z** Idiopathic inflammatory myopathy, unspecified

## Conclusions

In summary, the current results illustrate an increasing prevalence of myositis and highlight the complexity of this disease with regard to the above-mentioned comorbidities, especially neoplasms and psychiatric disorders.

## Data Availability

The datasets used and analysed during the current study are available from the corresponding author on reasonable request.

## Abbreviations

DM: Dermatomyositis
IIM: Idiopathic inflammatory myopathy
OM: Other myositis
PM: Polymyositis

## References

[1] Tsamis KI, Boutsoras C, Kaltsonoudis E (2021). Clinical features and diagnostic tools in idiopathic inflammatory myopathies. Critical Reviews in Clinical Laboratory Sciences.

[2] Casal-Dominguez M, Pinal-Fernandez I, Pak K (2021). Performance of the 2017 EULAR/ACR classification criteria for inflammatory myopathies in patients with myositis-specific autoantibodies. Arthritis & Rheumatology.

[3] Lundberg IE, Fujimoto M, Vencovsky J (2021). Idiopathic inflammatory myopathies. Nature Reviews Disease Primers.

[4] Parker MJS, Oldroyd A, Roberts ME (2019). The performance of the European League Against Rheumatism/American College of Rheumatology idiopathic inflammatory myopathies classification criteria in an expert-defined 10 year incident cohort. Rheumatology (Oxford).

[5] Leclair V, Lundberg IE (2018). New myositis classification criteria—What we have learned since Bohan and Peter. Current Rheumatology Reports.

[6] Oddis CV, Aggarwal R (2018). Treatment in myositis. Nature Reviews Rheumatology.

[7] Albrecht K, Huscher D, Callhoff J (2020). Trends in idiopathic inflammatory myopathies: Cross-sectional data from the German National Database. Rheumatology International.

[8] Korsten P, Rademacher J-G, Seitz CS (2019). Interdisziplinäre Fallkonferenzen als Chance für Myositis-Patienten?. Nervenheilkunde.

[9] Seiffert M, Brunner FJ, Remmel M (2020). Temporal trends in the presentation of cardiovascular and cerebrovascular emergencies during the COVID-19 pandemic in Germany: An analysis of health insurance claims. Clinical Research in Cardiology.

[10] Diers J, Acar L, Baum P (2021). Fewer operations for cancer in Germany during the first wave of COVID-19 in 2020—A cohort study and time-series analysis. Deutsches Ärzteblatt International.

[11] Meyer A, Meyer N, Schaeffer M, Gottenberg JE, Geny B, Sibilia J (2015). Incidence and prevalence of inflammatory myopathies: A systematic review. Rheumatology (Oxford).

[12] Bernatsky S, Joseph L, Pineau CA (2009). Estimating the prevalence of polymyositis and dermatomyositis from administrative data: Age, sex and regional differences. Annals of the Rheumatic Diseases.

[13] Carey IM, Banchoff E, Nirmalananthan N (2021). Prevalence and incidence of neuromuscular conditions in the UK between 2000 and 2019: A retrospective study using primary care data. PLoS ONE.

[14] Ohta A, Nagai M, Nishina M, Tomimitsu H, Kohsaka H (2014). Prevalence and incidence of polymyositis and dermatomyositis in Japan. Modern Rheumatology.

[15] Dobloug C, Garen T, Bitter H (2015). Prevalence and clinical characteristics of adult polymyositis and dermatomyositis; Data from a large and unselected Norwegian cohort. Annals of the Rheumatic Diseases.

[16] Tran TN, Steffey A, Caspard H (2013). FRI0447 Epidemiology of idiopathic inflammatory myopathies in England—A database analysis. Annals of the Rheumatic Diseases.

[17] Gonzalez-Mazon I, Sanchez-Bilbao L, Martin-Varillas JL (2021). Immune-related adverse events in patients with solid-organ tumours treated with immunotherapy: A 3-year study of 102 cases from a single centre. Clinical and Experimental Rheumatology.

[18] Nguyen T, Maria ATJ, Ladhari C (2022). Rheumatic disorders associated with immune checkpoint inhibitors: What about myositis? An analysis of the WHO's adverse drug reactions database. Annals of the Rheumatic Diseases.

[19] Seki M, Uruha A, Ohnuki Y (2019). Inflammatory myopathy associated with PD-1 inhibitors. Journal of Autoimmunity.

[20] Tanboon J, Uruha A, Stenzel W, Nishino I (2020). Where are we moving in the classification of idiopathic inflammatory myopathies?. Current Opinion in Neurology.

[21] Mariampillai K, Granger B, Amelin D (2018). Development of a new classification system for idiopathic inflammatory myopathies based on clinical manifestations and myositis-specific autoantibodies. JAMA Neurology.

[22] Hill CL, Zhang Y, Sigurgeirsson B (2001). Frequency of specific cancer types in dermatomyositis and polymyositis: A population-based study. Lancet.

[23] Yang Z, Lin F, Qin B, Liang Y, Zhong R (2015). Polymyositis/dermatomyositis and malignancy risk: A metaanalysis study. Journal of Rheumatology.

[24] Li Y, Jia X, Sun X (2021). Risk factors for cancer-associated myositis: A large-scale multicenter cohort study. International Journal of Rheumatic Diseases.

[25] Lilleker JB, Vencovsky J, Wang G (2018). The EuroMyositis registry: An international collaborative tool to facilitate myositis research. Annals of the Rheumatic Diseases.

[26] Opinc AH, Makowska JS (2022). Update on malignancy in myositis-well-established association with unmet needs. Biomolecules.

[27] Oldroyd AGS, Allard AB, Callen JP (2021). A systematic review and meta-analysis to inform cancer screening guidelines in idiopathic inflammatory myopathies. Rheumatology (Oxford).

[28] Achtman J, Kling MA, Feng R, Okawa J, Werth VP (2016). A cross-sectional study of untreated depression and anxiety in cutaneous lupus erythematosus and dermatomyositis. Journal of the American Academy of Dermatology.

[29] Naveen R, Rathore U, Agarwal V, Gupta L (2021). Characteristics and outcomes of overlap myositis: A comparative multigroup cohort study in adults from the MyoCite cohort. Rheumatology International.

[30] Doyle TJ, Dhillon N, Madan R (2018). Rituximab in the treatment of interstitial lung disease associated with antisynthetase syndrome: A multicenter retrospective case review. Journal of Rheumatology.

[31] Fasano S, Gordon P, Hajji R, Loyo E, Isenberg DA (2017). Rituximab in the treatment of inflammatory myopathies: A review. Rheumatology (Oxford).

[32] Moghadam-Kia S, Oddis CV, Aggarwal R (2017). Modern therapies for idiopathic inflammatory myopathies (IIMs): Role of biologics. Clinical Reviews in Allergy and Immunology.

[33] Schilling, M. K. R.: *Kodierleitfaden Neurologie*. https://dgn.org/wp-content/uploads/2020/05/Kodierleitfaden_Neurologie_2020_final.pdf

[34] Organization WH. *ICD-11*. https://icd.who.int/browse11/l-m/en

[35] Oldroyd A, Chinoy H (2018). Recent developments in classification criteria and diagnosis guidelines for idiopathic inflammatory myopathies. Current Opinion in Rheumatology.

